# Clinical Effectiveness of Pre-hospital and In-hospital Optimized Emergency Care Procedures for Patients With Acute Craniocerebral Trauma

**DOI:** 10.3389/fsurg.2021.830571

**Published:** 2022-01-17

**Authors:** Lili Wang, Rong Wu

**Affiliations:** ^1^Department of Emergency, The Nanhua Affiliated Hospital, Hengyang Medical College, University of South China, Hengyang, China; ^2^Department of Outpatients, The Nanhua Affiliated Hospital, Hengyang Medical College, University of South China, Hengyang, China

**Keywords:** acute craniocerebral injury, pre-hospital and in-hospital full optimization, emergency care, inflammatory factors, quality of life

## Abstract

Acute craniocerebral injury is a common traumatic disease in clinical practice, characterized by rapid changes in condition and a high rate of death and disability. Early and effective emergency care throughout the pre-hospital and in-hospital period is the key to reducing the rate of death and disability and promoting the recovery of patients. In this study, we conducted an observational study of 130 patients with acute craniocerebral injury admitted between May 2020 and May 2021. Patients were randomly divided into a regular group and an optimization group of 65 patients each, with patients in the regular group receiving the conventional emergency care model and patients in the optimization group receiving the pre-hospital and in-hospital optimal emergency care process for intervention. In this study, we observed and compared the time taken to arrive at the scene, assess the condition, attend to the patient and provide emergency care, the success rate of emergency care within 48 h, the interleukin-6 (IL-6), interleukin-8 (IL-8), and intercellular adhesion molecule-1 (ICAM-1) after admission and 1 day before discharge, the National Institute of Health Stroke Scale (NIHSS) and the Short Form 36-item Health Survey (SF-36) after resuscitation and 1 day before discharge, and the complications of infection, brain herniation, central hyperthermia, and electrolyte disturbances in both groups. We collected and statistically analyzed the recorded data. The results showed that the time taken to arrive at the consultation site, assess the condition, receive the consultation, provide first aid was significantly lower in the optimized group than in the regular group (*P* < 0.05); the success rate of treatment was significantly higher in the optimized group than in the regular group (*P* < 0.05). In both groups, IL-6, IL-8, and ICAM-1 decreased on the day before discharge compared with the day of rescue, with the levels of each index lower in the optimization group than in the regular group (*P* < 0.05); the NIHSS scores decreased and the SF-36 scores increased on the day before discharge compared with the successful rescue in both groups, with the NIHSS scores in the optimization group lower than in the regular group and the SF-36 scores higher than in the control group (*P* < 0.05). The overall complication rate in the optimization group was significantly lower than that in the regular group (*P* < 0.05). This shows that optimizing pre-hospital and in-hospital emergency care procedures can significantly shorten the time to emergency care for patients with acute craniocerebral injury, increase the success rate, reduce inflammation, improve neurological function and quality of life, reduce the occurrence of complications, and improve patient prognosis.

## Introduction

Acute craniocerebral injuries are mainly caused by traffic accidents, accidental injuries and violence. With the contemporary development of industry, construction and transportation in China, the incidence of acute craniocerebral injury has increased significantly, and traumatic acute craniocerebral injury is the most common (1, 2). Epidemiological studies have shown that in recent years about 28.7% (about 370 million) of the population in China has been affected each year, and the incidence of acute craniocerebral injury has continued to increase, with heavy craniocerebral injury accounting for about 20% and death in nearly 10% of patients with the disease (3, 4). In acute craniocerebral injury, if the injury involves only the scalp and skull alone, the patient's prognosis is more likely to be good after aggressive treatment (5). In contrast, the condition of patients with brain injury is often complex and rapidly developing, and if the injury is not treated promptly and effectively, it often leads to more serious adverse consequences, threatening the life of the patient and causing injury-related death and permanent disability (6, 7).

The key to the treatment of acute craniocerebral injury is timing. Taking effective emergency measures as early as possible, making the chain of pre-hospital resuscitation, in-hospital emergency care, and surgical implementation more compact, with a clearer division of labor and more orderly steps, is the key to improving the effectiveness of emergency care (8, 9). The traditional emergency method for patients with acute severe craniocerebral injury is to consult and treat them in separate departments, which cannot achieve good articulation between various departments and delays the treatment time, which is not conducive to improving the survival rate of patients (10). Pre-hospital and in-hospital whole process optimization care further evolves on the traditional emergency mode, effectively perfecting the pre-hospital rescue network and trauma rescue mode, and making the whole emergency process procedural and standardized (11, 12). Currently this model of care has been widely used clinically in the emergency care of acute cerebral infarction (13), acute myocardial infarction (14) and other clinical emergencies, and good results have been achieved. In view of this, the present study applied the pre-hospital and in-hospital optimal nursing procedure to the emergency treatment of patients with acute craniocerebral injury, and used the emergency-related indexes, the success rate of treatment, the level of inflammatory factors in different periods, the neurological function and quality of life in different periods, and the occurrence of complications as the observation indexes to explore the effect of the pre-hospital and in-hospital optimal nursing procedure in the application of acute craniocerebral injury.

## Materials and Methods

### Materials

One hundred and thirty cases of acute craniocerebral injury admitted to our hospital from May 2020 to May 2021 were selected as the study subjects. Patients were coded in order of admission for patients sorted from 1 to 130, and the enrolled patients were randomly divided into 65 cases each in the regular group and the optimized group according to the random number table method. Patients in the regular group received the conventional emergency care model and patients in the optimization group received the pre-hospital and in-hospital full optimization of emergency care procedures for intervention. General data such as age, gender, cause of injury, type of cranial injury and degree of injury [assessed according to the Glasgow Coma Scale (15), where 3–8 were classified as severe, 9–12 as moderate, and 13–15 as mild] were collected and compared between the two groups, and the differences were not statistically significant (*P* > 0.05, Table 1) and were comparable.

**Table 1:** Comparison of baseline demographic information for the regular group and optimization group.

**Data**		**Regular group (*n* = 65)**	**Optimization group (*n* = 65)**	**χ^2^ value**	***P-*value**
Gender (*n*, %)	Male	39 (60.00)	41 (63.08)	0.130	0.718
	Female	26 (40.00)	24 (36.92)		
Age (*n*, %)	18~25 years	12 (18.46)	14 (21.54)	0.648	0.885
	25~40 years	24 (36.93)	25 (38.46)		
	40~55 years	18 (27.69)	18 (27.69)		
	55~65 years	11 (16.92)	8 (12.31)		
Causes of injury (*n*, %)	Traffic accidents	27 (41.53)	25 (38.46)	0.301	0.960
	Drop	16 (24.62)	15 (23.08)		
	Hitting	15 (23.08)	17 (26.15)		
	Crush	7 (10.77)	8 (12.31)		
Degree of damage (*n*, %)	Mild	28 (43.08)	26 (40.00)	1.184	0.553
	Moderate	26 (40.00)	23 (35.38)		
	Severe	11 (16.92)	16 (24.62)		
Types (*n*, %)	Brain contusion and laceration	25 (38.46)	26 (40.00)	0.448	0.930
	Intracranial hematoma	20 (30.77)	18 (27.69)		
	Concussion	14 (21.54)	13 (20.00)		
	Skull base fracture	6 (9.23)	8 (12.31)		

### Inclusion Criteria

1. Age >18 years; 2. admission within 24 h of injury onset; 3. a clear history of trauma and a clear diagnosis of traumatic brain injury. 4. Patient and family understood the content of this study and voluntarily signed an informed consent form.

### Exclusion Criteria

1. Patients who died in pre-hospital emergencies; 2. patients with other serious injuries in combination; 3. patients with previous neurological or limb movement dysfunction; 4. patients with a combined history of psychiatric disorders, hematological disorders and cancer in various organ systems. 5. Multiple trauma such as combined organ damage, fractures in other areas, severe shock, etc.

### Care Methods

Patients in the regular group adopted the traditional emergency care model, as follows: after patients were transferred to the hospital by family members or lower hospitals, or by emergency vehicles, health care workers promptly assessed the patients' clinical symptoms, signs and past history to understand them. Patients were resuscitated according to the acute craniocerebral injury emergency procedures, including active management of trauma, routine oxygenation, monitoring of signs, and symptomatic treatment, while the imaging department was contacted to improve imaging and assess the condition. Patients who required surgical treatment were treated preoperatively and finally admitted to the clinical operating theater or ward, and their families were contacted afterwards to educate them on the precautions to take in their daily self-care.

Patients in the optimization group adopted a pre-hospital and in-hospital whole process of optimizing emergency care, with the following details: 1. Construct a care pathway team consisting of senior emergency department nurses, emergency physicians and 3–4 paramedics. Develop optimal emergency care protocols throughout the process by searching the literature and seeking input from primary care providers. All members of the Emergency Department were trained by members of the team to regularly check and ensure that emergency equipment was in good standby condition. 2. Pre-departure optimization: the ambulance was dispatched within 3–5 min after receiving the call for dispatch. The experienced and professional ambulance driver will try to reach the scene of resuscitation in the shortest possible time according to the precise navigation system. The ambulance attendant keeps in touch with the patient's family or personnel at the scene by telephone to guide pre-hospital emergency care (16, 17). 3. On-site emergency optimization: On arrival, a brief assessment of the patient's condition was carried out and appropriate care measures were quickly developed and tailored to the patient's specific situation. Ensure that the head, trunk and limbs were at the same level during transport. 4. Optimization of transfer rescue: healthcare workers should maintain gentle movements during transport and monitor the patient's vital signs such as mental state, respiratory rate, and blood pressure in real time during transport. Communicate with the escort to understand the basic situation of the patient, as well as informing the escort of the treatment and care after admission. After a thorough assessment of the patient's condition, the hospital departments were contacted in a timely manner according to their specific conditions, and urged them to make various emergency preparations. 5. Optimization of in-hospital emergency care: patients were admitted to hospital and a simple and rapid initial judgement was made by the emergency physician, with nursing staff completing a series of procedures such as placement, administering oxygen, advising on intravenous circulation, retaining blood specimens, assisting with electrocardiography, and taking medical histories according to medical advice. The first aid measures were tailored to the patient's specific situation. If patients show symptoms such as headache, restlessness or high fever, the health care provider should analyse the cause of the symptoms and take different measures to treat them. Patients who required emergency surgery should be assisted in their preoperative preparation (18–20). 6. Optimize health education: After the completion of emergency treatment, nursing staff explained in detail to the patient's family about health knowledge such as nursing precautions and self-care capabilities, and instructed the family to help the patient carry out neurological function rehabilitation exercises and motor rehabilitation training.

### Observation Indicators

#### Emergency Treatment Time

The indicators related to emergency care such as arrival time (the time between receiving the emergency call and the arrival of the emergency vehicle at the scene), assessment time (the time taken by the medical personnel to make a preliminary judgment on the patient's condition after reaching the emergency scene), receiving time (the time between arriving at the emergency scene and taking the patient to the hospital), emergency treatment time (the time between arriving at the emergency room of the emergency department and the end of resuscitation) were counted and compared between the two groups.

#### Resuscitation Within 48 h

The success rate of emergency treatment within 48 h (21) was assessed in both groups, and the effect of treatment was classified into four grades: success, markedly effective, valid, and invalid, which was assessed according to the National Institute of Health Stroke Scale (NIHSS). Success was defined as patients with stable vital signs and complete resolution of clinical symptoms after resuscitation, with a reduction in NIHSS score of more than 90%. Markedly effective was defined as the patient's vital signs were stable after resuscitation, clinical symptoms were significantly improved and NIHSS scores were reduced by 51–90%. Valid means that the patient's vital signs were generally stable after resuscitation, and the patient's condition has improved, and the NIHSS score has been reduced by 21–50%. Invalid means that the patient's vital signs remained unstable after resuscitation, and his condition did not improve or even worsened, and the NIHSS score was reduced by no more than 20%. Resuscitation success rate = number of cases (success + markedly effective + valid)/total number of effective cases × 100%.

#### Serum Inflammatory Factor Levels

Five milliliter of fasting venous blood was collected from patients 1 day after admission and 1 day before discharge, and the supernatant was centrifuged at 3,000 r/min for 10 min and placed at −80°C for testing. The serum levels of inflammatory factors such as interleukin-6 (IL-6), interleukin-8 (IL-8), and intercellular adhesion molecule-1 (ICAM-1) were measured using an enzyme-linked immunoassay kit (ELISA) supplied by Wuhan Fien Biotechnology Co.

#### Neurological Function and Quality of Life

Patients were assessed on the NIHSS scale after resuscitation and 1 day before discharge, with a total score of 0–42, with higher scores indicating more severe neurological impairment. The Short Form 36-item Health Survey (SF-36) was used to assess the quality of life of the two groups of patients. The scale consists of eight components: physical function, physical function, physical pain, general health, vitality, social practice, emotional function, and mental health. The scale was scored on a scale of 0–100, with higher scores indicating better quality of life.

#### Complications

The occurrence of complications such as infection, brain herniation, central hyperthermia, and electrolyte disturbance were counted and compared between the two groups.

### Statistical Methods

SPSS22.0 software was used for statistical analysis of the data, and Prism8.0 was used to draw the pictures. The measurement data conforming to normal distribution were expressed as mean ± standard deviation (mean ± SD), and *t*-test was performed for comparison between two groups; the count data were expressed as number of cases and composition ratio (*n*, %), and χ^2^ test was used for comparison between groups. *P* < 0.05 indicated statistically significant difference.

## Results

### Comparison of First Aid-Related Indicators Between the Two Groups

**Figure 1:**
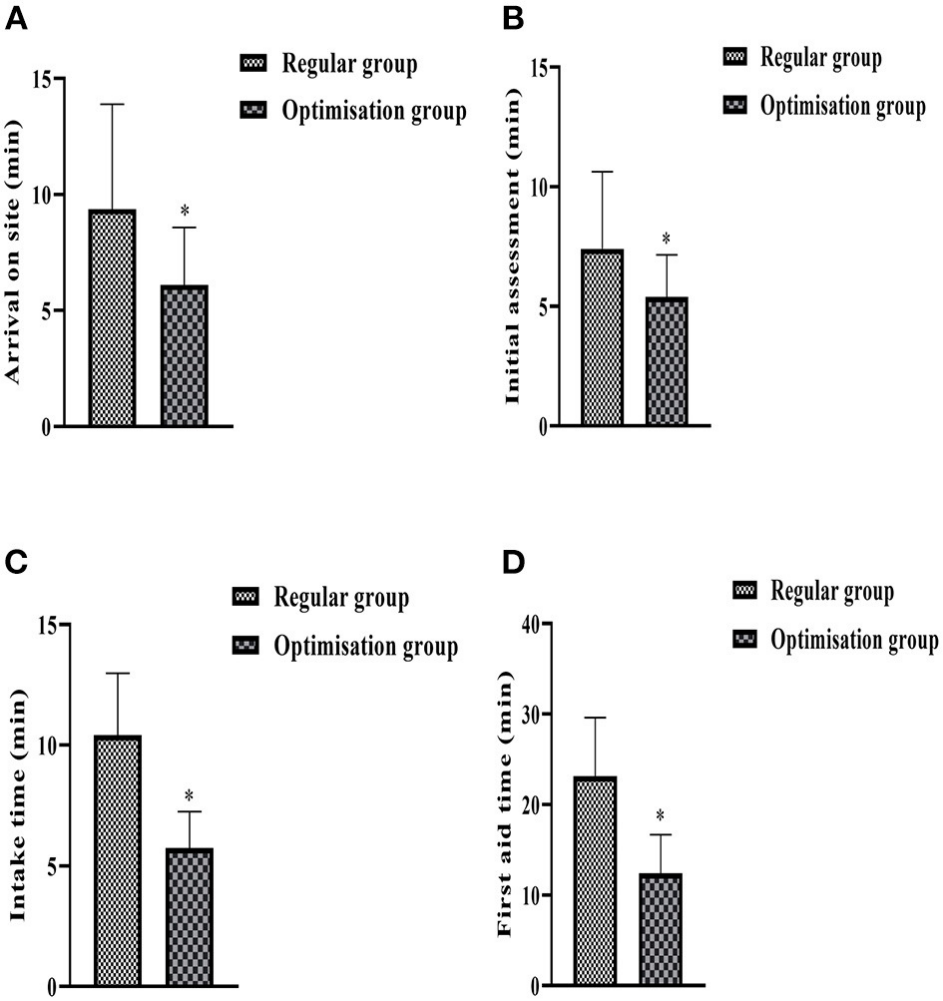
Histogram comparing the indicators related to the two groups of first aid. **(A)** Shows the time spent on arrival at the scene. **(B)** Shows the time taken to assess the condition on site. **(C)** Shows the time taken to receive a consultation after admission to hospital. **(D)** Shows the time taken for first aid. *Denotes comparison with regular group, *P* < 0.05.

The time spent on arrival at the scene, initial assessment, intake time and first aid in the two groups was counted, and the result of the comparison was that the time spent on arrival at the scene (Figure 1A), initial assessment (Figure 1B), intake time (Figure 1C), and first aid (Figure 1D) in the optimized group was lower than that in the regular group, and the difference was statistically significant (*P* < 0.05) (Figure 1).

### Comparison of the Success Rate of First Aid Within 48 h Between the Two Groups

**Figure 2:**
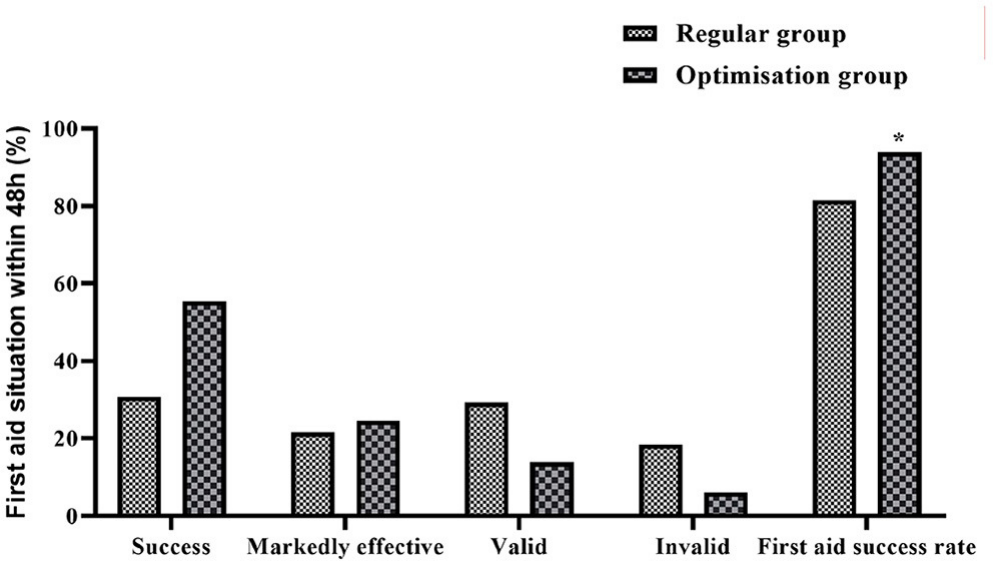
Histogram comparing the success rate of resuscitation within 48 h between the two groups. *Denotes comparison with regular group, *P* < 0.05.

The first aid situation of the two groups within 48 h was counted. In the regular group, 20 cases were successfully treated, 14 cases were markedly effective, 19 cases were effective, 12 cases were ineffective, and 53 cases (81.54%) were successful in first aid. In the optimization group, 36 cases were successfully treated, 16 cases were markedly effective, 9 cases were effective, 4 cases were ineffective, and 61 cases (93.85%) were successful in first aid. The results of the comparison showed that the success rate of first aid in the optimization group was significantly higher than that of the regular group, and the difference was statistically significant (*P* < 0.05) (Figure 2).

### Comparison of Serum Inflammatory Factor Levels Between the Two Groups at Different Times

**Figure 3:**
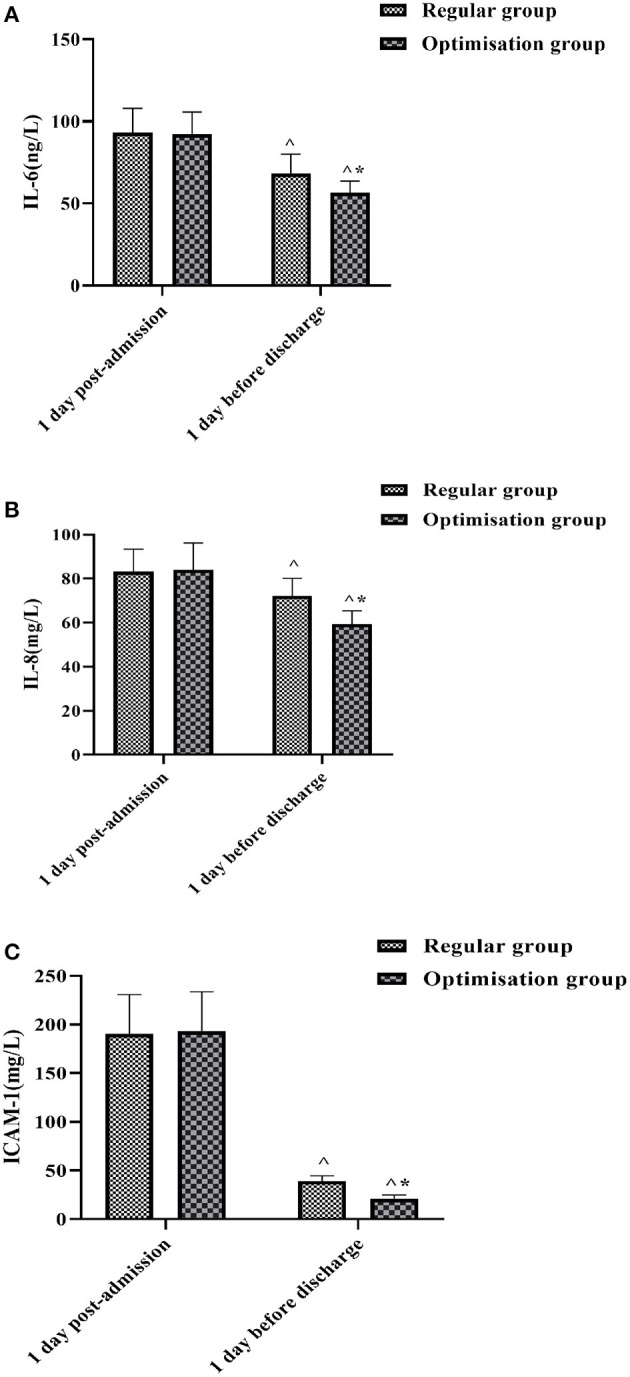
Histogram comparing IL-6, IL-8, and ICAM-1 between the two groups at different times. **(A)** Shows the comparison of IL-6 levels. **(B)** Shows the comparison of IL-8 levels. **(C)** Shows the comparison of ICAM-1 levels. ^∧^Denotes comparison with the same group 1 day post-admission, *P* < 0.05. *Denotes comparison with the regular group, *P* < 0.05.

Blood specimens were collected from both groups on the first day after admission and the first day before discharge and their serum inflammatory factor levels were measured. The results showed that the differences in serum IL-6, IL-8, and ICAM-1 levels between the two groups on the first day after admission were not statistically significant (*P* > 0.05). The levels of IL-6 (Figure 3A), IL-8 (Figure 3B), and ICAM-1 (Figure 3C) in both groups 1 day before discharge were significantly lower than those on admission, with the optimized group being lower than the regular group, and the differences were all statistically significant (*P* < 0.05).

### Comparison of Neurological Function and Quality of Life Scores Between the Two Groups at Different Times

**Figure 4:**
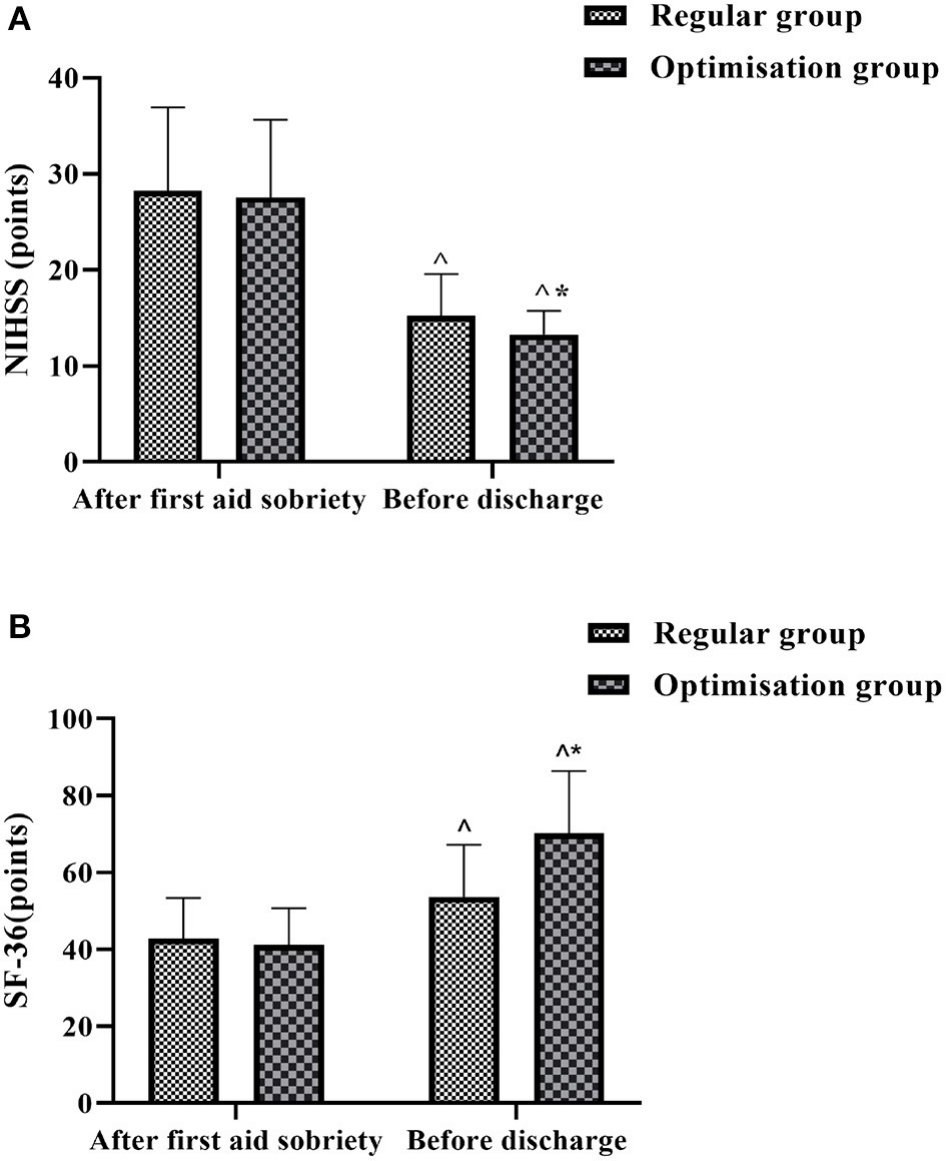
Histogram comparing NIHSS scores and SF-36 scores between the two groups at different times. **(A)** Shows the comparison of NIHSS scores. **(B)** shows the comparison of SF-36 scores. ^∧^Denotes comparison with the same group after first aid soberiety, *P* < 0.05. *Denotes comparison with the regular group, *P* < 0.05.

The NIHSS and SF-36 were used to assess the neurological function and quality of life of the patients after resuscitation and before discharge, respectively. The difference between the NIHSS and SF-36 scores after resuscitation and awakening was not statistically significant (*P* > 0.05). In both groups, the pre-discharge NHISS scores (Figure 4A) decreased compared to the post-resuscitation awakening and the SF-36 scores (Figure 4B) increased compared to the post-resuscitation awakening, with the pre-discharge NHISS scores in the optimization group being lower than those in the regular group and the SF-36 scores being higher than those in the regular group, both with statistically significant differences (*P* < 0.05) (Figure 4).

### Comparison of the Occurrence of Complications in the Two Groups

**Figure 5:**
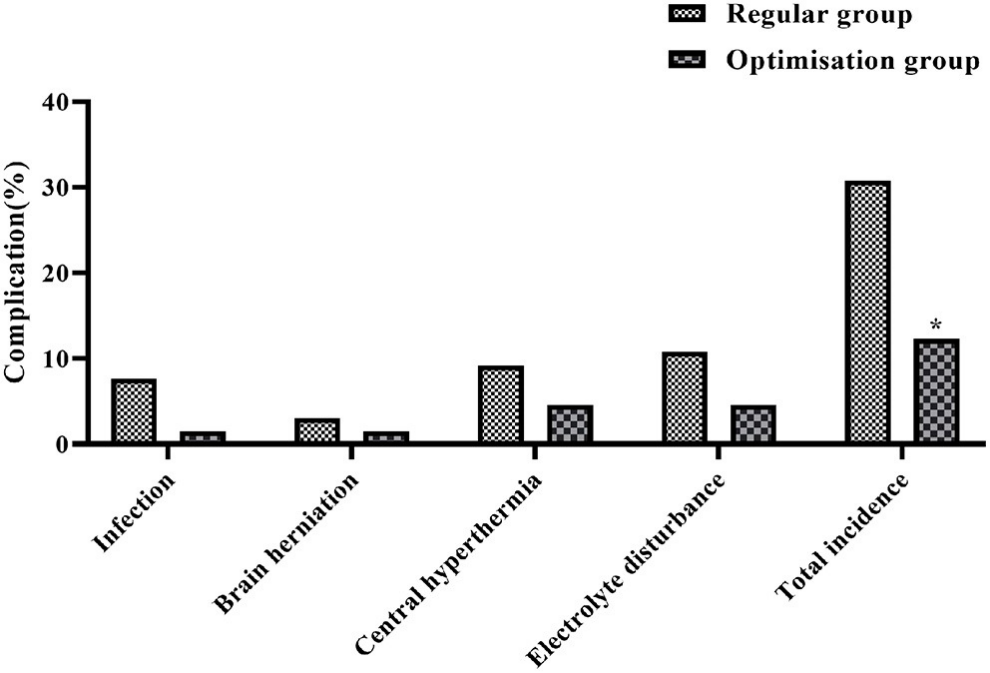
Histogram comparing the incidence of complications between the two groups. *Denotes comparison with regular group, *P* < 0.05.

The overall incidence of complications such as infection (5 cases), brain herniation (2 cases), central hyperthermia (6 cases), and electrolyte disturbances (7 cases) in the regular group was 30.77% (20/65). In the optimization group, the overall complication rate of infection (1 case), brain herniation (1 case), central hyperthermia (3 cases), and electrolyte disturbances (3 cases) was 12.31% (20/65). The results of the comparison showed that the overall incidence of complications in the optimization group was lower than that in the regular group, with a statistically significant difference (*P* < 0.05) (Figure 5).

## Discussion

### The Need for Timely Treatment of Acute Craniocerebral Injury

Acute craniocerebral injury is usually caused by traffic accidents or accidental injuries. After the injury, patients may experience a series of changes in the brain such as microcirculatory obstruction, cerebral blood circulation dysfunction, and rapid increase in intracerebral pressure, which may lead to respiratory depression, blurred consciousness, cerebral tissue ischemia, and electrolyte disorders if not rescued in time (22, 23). Patients with severe injuries are susceptible to rapid changes and complications that make treatment more difficult, so they need to be treated promptly and effectively after acute craniocerebral injury (24). The success of acute craniocerebral injury treatment is closely related to the severity of the injury, the timeliness of emergency care and treatment, the sophistication of the equipment and the professionalism of the medical staff (25). Pre-hospital and in-hospital optimal care is a combination of pre-hospital emergency care, emergency department resuscitation and in-hospital treatment, and is a nursing procedure developed on the basis of traditional emergency care (26). We have applied pre-hospital and in hospital optimized emergency care to the emergency treatment of patients with acute craniocerebral injury. By optimizing the five components of post-acute discharge, on-site emergency care, transport and rescue, in-hospital emergency care and health education, we have achieved a seamless integration of all components, greatly improving the efficiency of emergency care for patients with acute craniocerebral injury and gaining precious time for their treatment.

### Impact of Pre-hospital and In-hospital Optimal Care Procedures Throughout on Indicators Related to Life-Saving Treatment

The results of the study showed that the time taken by the optimization group to reach the scene of the accident, the initial assessment of the condition, the time taken to receive treatment and in-hospital emergency care was significantly lower than that of the regular group (*p* < 0.05). Optimize every measure and operation from the dispatch of the ambulance to the completion of the handover, and regularly check the adequacy and perfection of the materials and equipment required for the first aid of acute brain injury, and dispatch the ambulance as soon as the call is received. When going to the accident site and transferring the ambulance, the most suitable route is chosen by combining the driver's rich experience and the accurate navigation system; before reaching the accident site, the medical personnel get in touch with the people around the patient, have a preliminary understanding of the basic situation of the patient through the telephone, and guide him/her to carry out scientific treatment, and at the same time prepare the relevant equipment and drugs according to the basic situation of the patient, and work out with the doctor The initial resuscitation plan, which can save a lot of time in on-site emergency care (27). On the way back to the hospital, the patient's vital signs are closely monitored, the patient's condition is comprehensively assessed, and the corresponding department is contacted to prepare for consultation and consultation according to the trend of changes in the patient's condition, so that pre-hospital emergency and in-hospital emergency are seamlessly connected and the problem of pre-hospital and in-hospital disconnection is solved, which can also buy time for effective in-hospital emergency care for the patient (28). Therefore, the arrival time, initial assessment time, reception time and in-hospital resuscitation time of acute craniocerebral injury patients in the optimized group were all significantly reduced, suggesting that the optimized pre-hospital and in-hospital care procedure can effectively ensure that patients can be effectively treated within a shorter period of time and improve the efficiency of resuscitation.

### Effect of Pre-hospital and In-hospital Optimized Care Procedures on the Success Rate of Emergency Care and Complication Rates

The results of the study showed that the success rate of emergency care within 48 h was significantly higher in the optimization group than in the regular group (*P* < 0.05). In the conventional resuscitation mode, although the rescue facilities are perfect and the medical level is better guaranteed, there is poor articulation between pre-hospital and in-hospital resuscitation, and in a panic emergency situation, the medical and nursing staff do not cooperate with the steps tightly enough, which easily causes the patient to miss the best time for rescue and treatment, resulting in deterioration of the condition and thus serious complications such as brain herniation and infection, and the patient's prognosis is poor (29). The pre-hospital and in-hospital optimization process saves a great deal of time through the optimization of post-visit trips, on-site emergencies, and transport ambulances, which is important for preserving the time window for acute craniocerebral injury treatment and can have a direct impact on the outcome of the patient. In addition, the overall complication rate in the optimization group was significantly lower than in the regular group (*P* < 0.05), which is probably due to the fact that the pre-hospital and in-hospital optimization process, with its scientific, effective, and timely care measures, has greatly improved the efficiency of the patients' follow-up treatment, thus reducing the possibility of complications.

### Effect of Pre-hospital and In-hospital Optimal Care Procedures on Patients' Serum Inflammatory Factors, Neurological Function, and Quality of Life

The inflammatory response after acute craniocerebral injury is a major component of the pathological process and a leading factor in secondary brain injury after craniocerebral injury (30). After craniocerebral injury, the body can secrete and release large amounts of IL-6, IL-8, and other inflammatory factors into the blood, causing local vasodilatation and increased permeability, and extravasation into the plasma leading to the formation of tissue vasogenic oedema (31). In addition, ICAM-1 plays a role in the development of inflammation, and its elevated levels after craniocerebral injury can increase the adhesion of leukocytes to vascular endothelial cells, as well as directly into the surrounding tissue, exacerbating brain injury (32). By measuring serum IL-6, IL-8 and ICAM-1 levels in both groups at different times, we found that the levels of inflammatory factors in both groups decreased significantly in the first day before discharge compared with those at the time of admission, and the optimized group was lower than the regular group (*P* < 0.05). After timely and effective resuscitation, the patient's injuries and inflammatory response were well-controlled and the resuscitation was effective in helping to reduce the level of inflammatory factors. The results also showed that the NIHSS and SF-36 scores improved better in the optimization group than in the regular group, suggesting that the treatment of patients with acute craniocerebral injury under the guidance of a full optimized care program is more effective in improving neurological function and short-term quality of life.

## Conclusion

It can be seen that optimizing the pre-hospital and in-hospital emergency care process can significantly shorten the time to emergency care, increase the success rate, reduce the inflammatory response, improve neurological function, and quality of life, reduce the occurrence of complications and improve the prognosis of the patients.

## Data Availability Statement

The original contributions presented in the study are included in the article/supplementary material, further inquiries can be directed to the corresponding author.

## Ethics Statement

The studies involving human participants were reviewed and approved by the Nanhua Affiliated Hospital, Hengyang Medical College, University of South China. The patients/participants provided their written informed consent to participate in this study.

## Author Contributions

LW was mainly responsible for the formulation of the entire study and the inclusion of samples. RW was mainly responsible for the detection of results, statistics of data, and writing of the paper. All authors contributed to the article and approved the submitted version.

## Conflict of Interest

The authors declare that the research was conducted in the absence of any commercial or financial relationships that could be construed as a potential conflict of interest.

## Publisher's Note

All claims expressed in this article are solely those of the authors and do not necessarily represent those of their affiliated organizations, or those of the publisher, the editors and the reviewers. Any product that may be evaluated in this article, or claim that may be made by its manufacturer, is not guaranteed or endorsed by the publisher.
